# Associations between female lung cancer risk and sex steroid hormones: a systematic review and meta-analysis of the worldwide epidemiological evidence on endogenous and exogenous sex steroid hormones

**DOI:** 10.1186/s12885-021-08437-9

**Published:** 2021-06-10

**Authors:** Hui Zeng, Zhuoyu Yang, Jiang Li, Yan Wen, Zheng Wu, Yadi Zheng, Yiwen Yu, Yongjie Xu, Shugeng Gao, Fengwei Tan, Ni Li, Qi Xue, Jie He

**Affiliations:** 1 Department of Thoracic Surgery, National Cancer Center/National Clinical Research Center for Cancer/Cancer Hospital, Chinese Academy of Medical Sciences and Peking Union Medical College, 17 Panjiayuan Nanli, Chaoyang District, Beijing, 100021 China; 2 Office of Cancer Screening, National Cancer Center/National Clinical Research Center for Cancer/Cancer Hospital, Chinese Academy of Medical Sciences and Peking Union Medical College, 17 Panjiayuan Nanli, Chaoyang District, Beijing, 100021 China

**Keywords:** Women, Lung cancer, Sex steroid hormones, Meta-analysis

## Abstract

**Background:**

Published findings suggest sex differences in lung cancer risk and a potential role for sex steroid hormones. Our aim was to perform a meta-analysis to investigate the effects of sex steroid hormone exposure specifically on the risk of lung cancer in women.

**Methods:**

The PubMed, MEDLINE, Web of Science, and EMBASE databases were searched. The pooled odds ratios (ORs) and 95% confidence intervals (95% CIs) for female lung cancer risk associated with sex steroid hormones were calculated overall and by study design, publication year, population, and smoking status. Sensitivity analysis, publication bias, and subgroup analysis were performed.

**Results:**

Forty-eight studies published between 1987 and 2019 were included in the study with a total of 31,592 female lung cancer cases and 1,416,320 subjects without lung cancer. Overall, higher levels of sex steroid hormones, both endogenous (OR: 0.92, 95% CI: 0.87–0.98) and exogenous (OR: 0.86, 95% CI: 0.80–0.93), significantly decreased the risk of female lung cancer by 10% (OR: 0.90, 95% CI: 0.86–0.95). The risk of lung cancer decreased more significantly with a higher level of sex steroid hormones in non-smoking women (OR: 0.88, 95% CI: 0.78–0.99) than in smoking women (OR: 0.98, 95% CI: 0.77–1.03), especially in Asia women (OR: 0.84, 95% CI: 0.74–0.96).

**Conclusions:**

Our meta-analysis reveals an association between higher levels of sex steroid hormone exposure and the decreased risk of female lung cancer. Surveillance of sex steroid hormones might be used for identifying populations at high risk for lung cancer, especially among non-smoking women.

**Supplementary Information:**

The online version contains supplementary material available at 10.1186/s12885-021-08437-9.

## Background

Lung cancer is the leading cause of cancer-related mortality worldwide, accounting for 22% of all cancer deaths [1, 2]. Prevention of lung cancer based on tobacco control has been widely implemented worldwide; however, approximately 25% of lung cancer cases worldwide still occur in never-smokers, especially in women [3–8]. Notably, many more lung cancer cases occur in Asian never-smokers than in Western never-smokers [4, 9]. However, due to the uncertain causes of lung cancer besides tobacco smoking, both the primary prevention of lung cancer and lung cancer screening strategy based on the identification of high-risk populations are difficult, especially among women and never-smokers.

Knowledge about etiological and clinical lung cancer characteristics has been acquired from studies involving mainly men because of its rarity in women until the 1970s. However, the incidence of lung cancer in women has increased in recent decades [10]. Unlike lung cancer in men, in addition to less smoking, more adenocarcinoma and good prognoses have been found in lung cancer in women [11–14]. The different features existing between the genders are still unexplained, suggesting the existence of some factors associated with female lung cancer in addition to the common risk factors, such as tobacco smoking [15].

Research on the effects of sex steroid hormones on lung cancer risk might explain the sex differences of lung cancer. Biological studies have reported the expression of sex steroid hormone receptors, including estrogen, progesterone and androgen in human bronchial and alveolar epithelia and in airway smooth muscle, by which the sex steroid hormones play roles. Additionally, the presence of sex-steroid-synthesizing enzymes, as components of local metabolism in lung parenchyma, may also be involved in the development of chronic respiratory diseases, such as lung cancer [16]. These observations above have suggested that sex steroid hormones may affect the pathogenesis of lung cancer and prompt epidemiological studies to explore the associations between the levels of sex steroid hormone exposure and the risk of lung cancer in women. Additionally, increasing epidemiological evidence has shown that the levels of sex steroid hormone exposure (e.g., indicated by age at menarche, age at menopause, parity, and hormone use), might have effects on the development of lung cancer in women but with generally inconsistent findings [6, 16–28]. Progestogens and estrogens are the main sex steroid hormone exposure in women, and according to different sources, progestogen and estrogen exposure can be roughly divided into exogenous and endogenous hormone exposure. Exogenous hormone exposure includes oral contraception (OC), use of hormone replacement therapy (HRT) and isoflavone intake from food. And endogenous hormone exposure includes younger age at menarche, older age at menopause, longer reproductive windows (only for postmenopausal women, calculated as the duration between age at menopause and age at menarche), longer menstrual cycle, pregnancy history, younger age at first pregnancy and multiple pregnancies.

The effects of sex steroid hormones on the risk of lung cancer in women are possibly influenced by study design, varied by population, and biased by tobacco smoking. To systematically analyze the associations between the levels of sex steroid hormone exposure and the risk of lung cancer in women, we conducted a meta-analysis and systematic review**.**

## Methods

The appendix to this manuscript is publicly shared in an online repository [29]. This quantitative review is reported based on the Preferred Reporting Items for Systematic Reviews and Meta-Analysis (PRISMA) extension statement for network meta-analysis [30].

### Search strategy and selection criteria

The PubMed, MEDLINE, Web of Science, and EMBASE databases were searched for articles published from January 1987 through December 2019 using the MeSH terms “menstruation,” “menopause,” “fertility,” “hormone,” “human,” and “lung cancer.” Potentially eligible studies were also sought regularly by computer-aided literature searches and manual searches of review articles. The detailed search strategy is shown in reference [29] (Supplementary Table S1).

In the present meta-analysis, full-text reviews were performed considering the following inclusion criteria: (1) the study reported lung cancer diagnosis along with pathological or clinical results, and (2) the study contain sex steroid hormones information in female. If data subsets were published in more than one article, only the one with the largest number of lung cancer cases was included. The exclusion criteria were as follows: (1) the study involved patients without primary lung cancer; (2) the study was a survival cohort study, cytotoxic molecular experiment, case report, review, or editorial; (3) the study contained data that could not be extracted or calculated from the original article; and (4) the study contain a duplicate population. All studies were stored in EndNote X9.

### Data extraction

Two investigators (Hui Zeng and Zhuoyu Yang) independently extracted the data through a standardized data collection form and reached a consensus on all items. When a disagreement occurred, consensus was reached through discussion between the authors or consultation with a reviewer. Therefore, similar analytical methods could be used across all studies, and we incorporated prospective studies and case-control studies including information about lung cancer patients.

The following information was extracted from each study: the first author, calendar year of publication, study population, study design, sample sizes, whether the study had a matched design and variables used for matching, how the information on exposure was obtained (self-administered questionnaire, face-to-face interview, medical records, etc.) and the indicators of sex steroid hormones (Supplementary Table S2; Supplementary Table S3) [29]. The indicators of higher levels of endogenous sex steroid hormone exposure include younger age at menarche, older age at menopause, longer reproductive windows (only for postmenopausal women, calculated as the duration between age at menopause and age at menarche), longer menstrual cycle, pregnancy history, younger age at first pregnancy and multiple pregnancies. Meanwhile, the indicators of higher levels of exogenous sex steroid hormone exposure include use of oral contraception (OC), use of hormone replacement therapy (HRT) and isoflavone intake from food (Supplementary Table S2) [29]. Due to the inconsistency of individual study definitions of high-level hormone exposure, our classification draws on the original article classification (Supplementary Table S2) [29]. ‘Ever-smokers’ was defined as having smoked more than 100 cigarettes in one’s lifetime. Otherwise, the cases were categorized as ‘non-smokers’. If no detailed description about smoking status was available, we adopted the original definition proposed by the authors.

### Quality assessment

The potential risk of bias and applicability of the included studies were assessed according to the Newcastle-Ottawa quality assessment scale. This scale comprises eight items that are classified into three domains, namely, selection, comparability and outcome. A study earning six or more stars was considered to be of high quality. The detailed process is shown in the reference [29].

### Statistical analysis

A fixed-effects or random-effects model was used to pool the data based on the Mantel–Haenszel method and DerSimonian and Laird method, respectively [31, 32]. These two models provide similar results when between-studies heterogeneity is absent; otherwise, the random-effects model is more appropriate. Heterogeneity between the studies was assessed by the *chi-squared Q* statistic (a higher number indicating more heterogeneity between studies) and *I*^*2*^ value (50% indicating heterogeneity), and *P* < 0.05 was considered to indicate statistical significance. Meta-regression and subgroup analyses were performed to quantify between-study heterogeneity, which were accounted for by publication year, study population, study design, and tobacco smoking status (non- and ever-smokers). We also performed a sensitivity analysis by examining changes in the results produced by the exclusion of each study. To assess publication bias, funnel plots (the natural logarithm of the OR and its standard error (SE)) were constructed. The circles correspond to the log OR from individual studies, and the diagonal lines show the expected 95% CI of the summary estimate. Furthermore, we performed a linear regression test of funnel plot asymmetry to evaluate more potential factors and obtained the results of Egger’s test to indicate publication bias.

All statistical analyses and graphs were conducted using RevMan (Review Manager statistical software, version 5.3), R software (software, version 3.6.2, https://www.r-project.org/) and OriginPro software (Origin Software, Inc., San Clemente, CA; version 9.6.5.169).

## Results

**Fig. 1:**
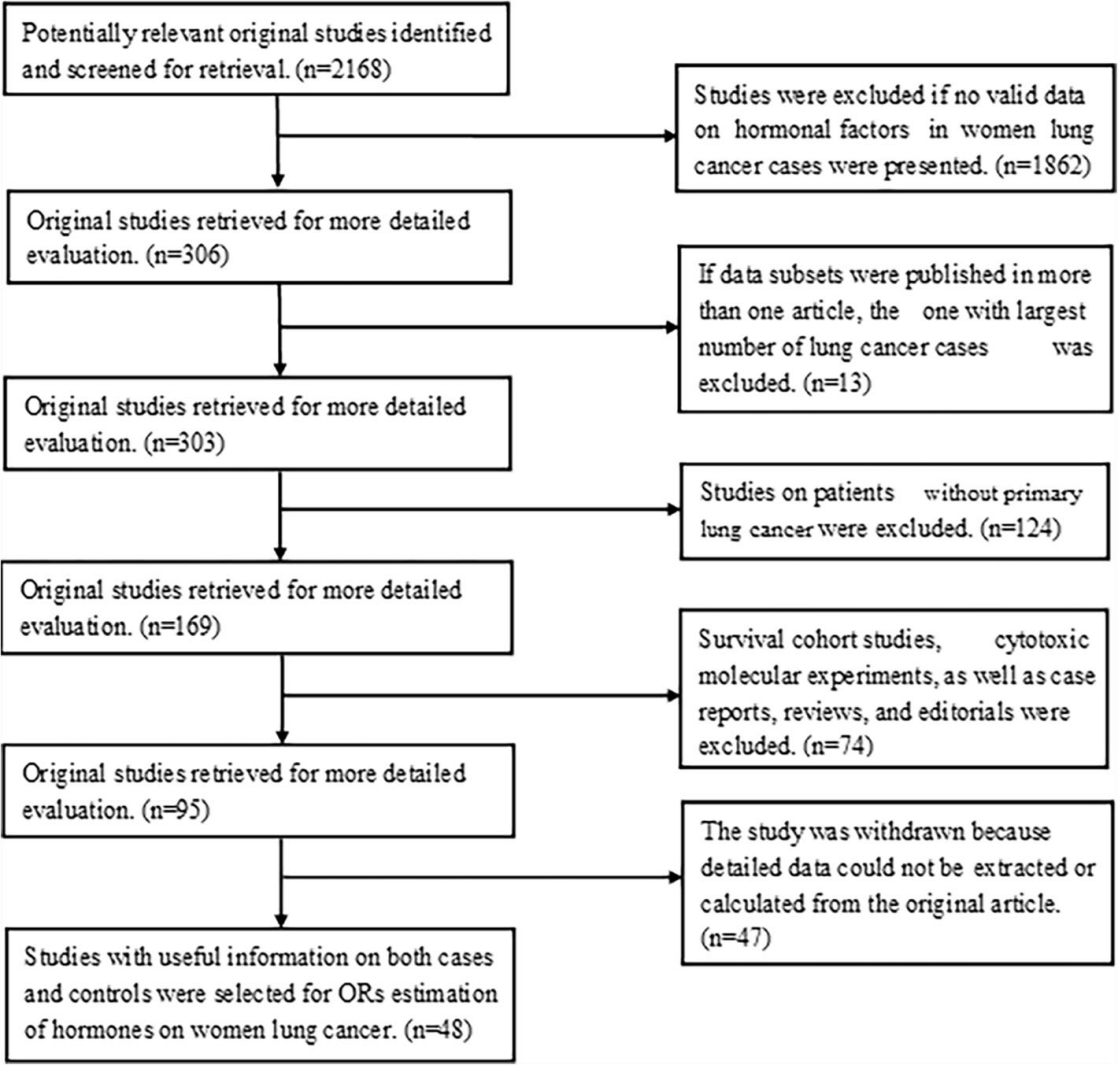
Flow-chart for Studies Selection

Overall, a total of 31,592 female lung cancer cases and 1,416,320 female subjects without lung cancer were included in the meta-analysis. Anonymized information of the individual participants was obtained from 48 studies (Fig. 1) conducted in ten countries, and approximately half of them (27 of 48) were in the West. Fourteen studies were prospective studies, and the rest were case-control studies. The characteristics of the 48 studies are listed in reference [29]. In terms of quality assessment, 41 studies obtained ≥7 stars, and the remaining 7 studies obtained 6 stars, indicating that the quality of the included studies was generally good (Supplementary Table S3) [29].

The present meta-analysis revealed that higher levels of sex steroid hormone exposure, both endogenous (OR: 0.92, 95% CI: 0.87–0.98) and exogenous sex steroid hormones (OR: 0.86, 95% CI: 0.80–0.93), significantly reduced the risk of lung cancer in women by 10% (OR: 0.90, 95% CI: 0.86–0.95). We also examined 14 studies (contained in our 48 studies) assessed both endogenous and exogenous sex steroid hormone exposure and found that higher levels of sex steroid hormone exposure reduced the risk of lung cancer in women by 11% (OR: 0.89, 95% CI: 0.84–0.95). (Supplementary Figure S1) [29] The effects of sex steroid hormones on the risk of lung cancer did not differ significantly by publication calendar year and study design (both *P* > 0.05) (Table 1; Supplementary Figure S2–3) [29].

**Table 1:** Associations between sex steroid hormones and the risk of female lung cancer

Variables	No. of studies	***x***^**2**^ for heterogeneity	Model selected	OR ^a^ (95% CI ^**b**^)	***P*** for OR heterogeneity
**Total**	48	1249.27	Random	**0.90 (0.86, 0.95)**	< .001
**Hormones resource**					**.190**
Endogenesis	26	679.81	Random	**0.92 (0.87, 0.98)**	< .001
Exogenesis	36	419.73	Random	**0.86 (0.80, 0.93)**	< .001
**Study design**					**.950**
Retrospective	34	573.55	Random	**0.90 (0.85, 0.96)**	< .001
Prospective	14	667.58	Random	**0.90 (0.81, 1.00)**	**<** .001
**Publication date**					**.370**
1987–2007	22	232.66	Random	**0.88 (0.82, 0.94)**	< .001
2008–2019	26	1016.31	Random	**0.92 (0.86, 0.99)**	**<** .001

^a^ *OR* Odds ratio, ^b^ *CI* Confidence interval

Table 2 showed that, in detail, younger age at menarche (OR: 0.93, 95% CI: 0.88–0.98), older age at menopause (OR: 0.79, 95% CI: 0.68–0.93), longer menstrual cycle (OR: 0.75, 95% CI: 0.60–0.94), use of OC (OR: 0.88, 95% CI: 0.81–0.96), use of HRT (OR: 0.89, 95% CI: 0.78–1.01) and higher isoflavone intake from food (OR: 0.73, 95% CI: 0.59–0.89) significantly or borderline significantly reduced the risk of lung cancer in women. Meanwhile, younger age at first pregnancy increased the risk (OR: 1.21, 95% CI: 1.05–1.39).

**Table 2:** Association between endogenous and exogenous sex steroid hormones and the risk of female lung cancer

Variables	No. of studies	***x***^**2**^ for heterogeneity	Model selected	OR ^**a**^ (95% CI ^**b**^)	***P*** for heterogeneity	Egger’s test (***P***-value)
**Total**	48	1249.27	Random	**0.90 (0.86, 0.95)**	< .001	–
**Indicators related to higher levels of endogenous sex steroid hormone exposure**	26	679.81	Random	**0.92 (0.87, 0.98)**	< .001	–
Younger age at menarche	18	26.51	Fixed	**0.93 (0.88, 0.98)**	.070	.5729
Older age at menopause	15	81.94	Random	**0.79 (0.68, 0.93)**	< .001	.3113
Longer reproductive windows	6	13.34	Random	0.90 (0.74, 1.10)	.020	.0566
Longer menstrual cycle	9	20.89	Random	**0.75 (0.60, 0.94)**	.007	.7938
History of pregnancy	15	37.47	Random	0.96 (0.84, 1.10)	< .001	.3856
Younger age at first pregnancy	15	69.48	Random	**1.21 (1.05, 1.39)**	< .001	.4527
Multiple pregnancies	20	92.20	Random	1.06 (0.94, 1.19)	< .001	.6782
**Indicators related to higher levels of exogenous sex steroid hormone exposure**	36	419.73	Random	**0.86 (0.80, 0.93)**	< .001	–
History of use of OC ^c^	22	72.67	Random	**0.88 (0.81, 0.96)**	< .001	.1380
History of use of HRT ^d^	25	327.30	Random	**0.89 (0.78, 1.01)**	< .001	.1725
Higher isoflavone intake from food	6	14.37	Random	**0.73 (0.59, 0.89)**	.010	.9897

^a^ *OR* Odds ratio; ^b^ *CI* Confidence interval, ^c^ *OC* Oral contraception, ^d^ *HRT* Hormone replacement therapy

**Fig. 2:**
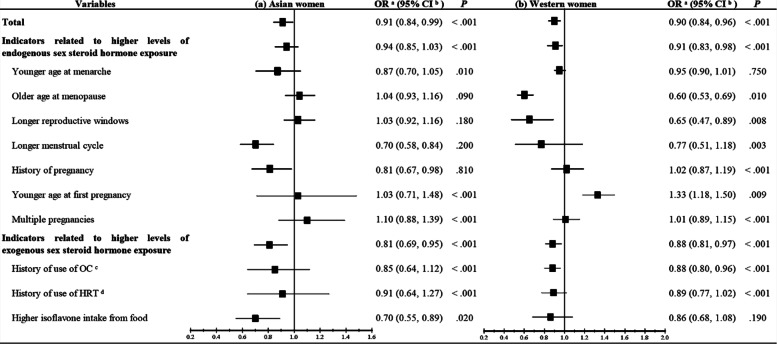
Forest plots for higher levels of sex steroid hormone exposure on the risk of lung cancer with **a** Asian women and **b** Western women. a. OR: Odds ratio; b. CI: Confidence interval; c. OC: Oral contraception; d. HRT: Hormone replacement therapy

For the marked variation in characteristics of lung cancer by population, the effects of sex steroid hormones on lung cancer in women were sub-group analyzed in Asian and Western women, respectively. Overall, in both Asian (OR: 0.91, 95% CI: 0.84–0.99) and Western (OR: 0.90, 95% CI: 0.84–0.96) women, higher levels of sex steroid hormone exposure significantly decreased the risk of lung cancer. The sub-analysis showed that for the indicators of levels of endogenous sex steroid hormone exposure longer menstrual cycle (OR: 0.70, 95% CI: 0.58–0.84) and history of pregnancy (OR: 0.81, 95% CI: 0.67–0.98) decreased the risk of lung cancer in Asian women, whereas older age at menopause (OR: 0.60, 95% CI: 0.53–0.69) and longer reproductive windows (OR: 0.65, 95% CI: 0.47–0.89) significantly decreased the risk of lung cancer in Western women. However, a younger age at first pregnancy increased the risk of lung cancer by 33% in the Western women (OR: 1.33, 95% CI: 1.18–1.50) (Fig. 2). For the indicators of levels of exogenous sex steroid hormone exposure, a higher isoflavone intake from food and use of OC decreased the risk of lung cancer in Asian and Western women by 30% (OR: 0,70, 95% CI: 0.55–0.89) and 12% (OR: 0.88, 95% CI: 0.80–0.96), respectively (Fig. 2).

**Fig. 3:**
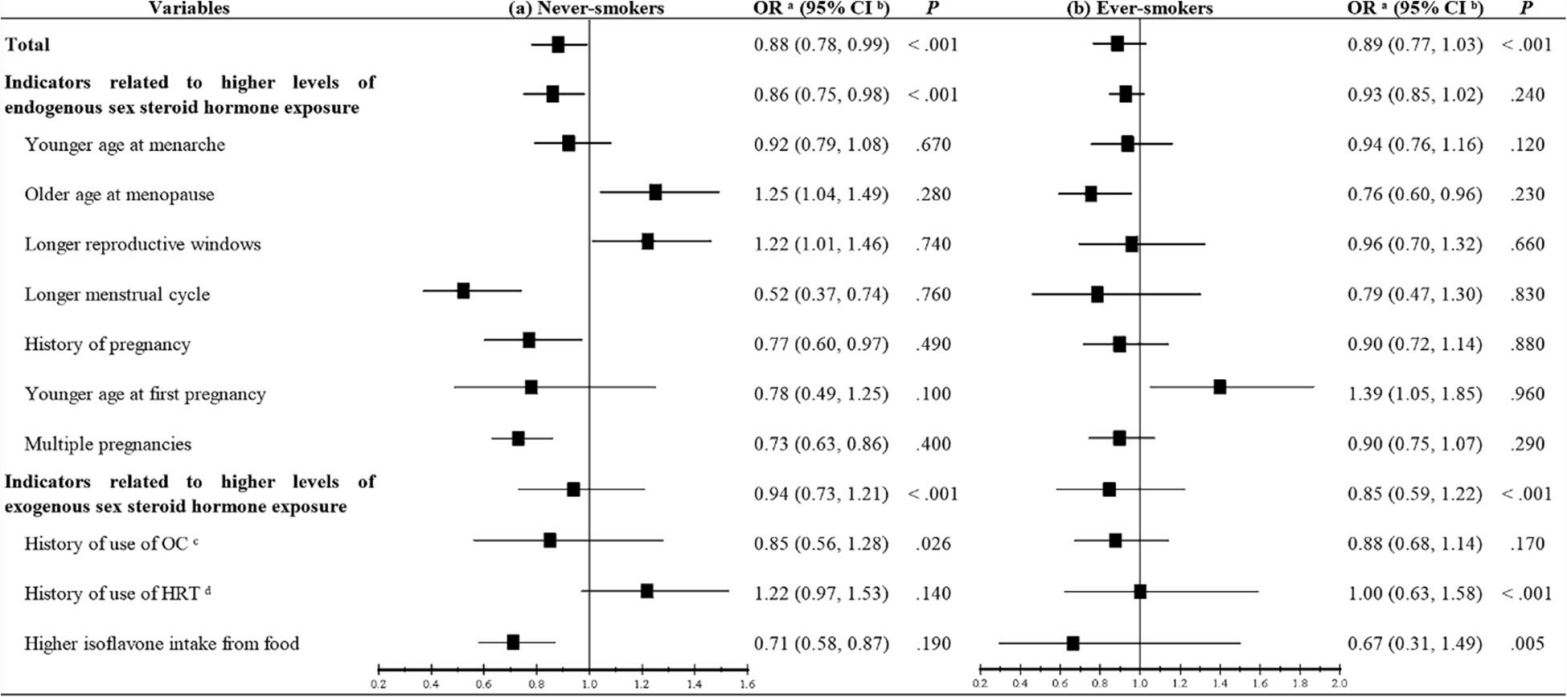
Forest plots for higher levels of sex steroid hormone exposure on the risk of female lung cancer with **a** never-smokers and **b** ever-smokers. a. OR: Odds ratio; b. CI: Confidence interval; c. OC: Oral contraception; d. HRT: Hormone replacement therapy

Seven case-control and three prospective studies that reported the association between sex steroid hormones and female lung cancer risk by cigarettes smoking were included in the sub-group analysis for smoking [33–42]. As shown in Fig. 3, higher levels of sex steroid hormone exposure, especially the endogenous sex steroid hormones (OR: 0.86, 95% CI: 0.75–0.98), decreased the risk of lung cancer in never-smokers more significantly (OR: 0.88, 95% CI: 0.78–0.99). Among never-smokers, older age at menopause (OR: 1.25, 95% CI: 1.04–1.49) and longer reproductive windows (OR: 1.22, 95% CI: 1.01–1.46) increased the risk of lung cancer. Meanwhile, a longer menstrual cycle, history of pregnancy, multiple pregnancies and higher isoflavone intake from food reduced the risk of female lung cancer by 48% (OR: 0.52, 95% CI: 0.37–0.74), 23% (OR: 0.77, 95% CI: 0.60–0.97), 27% (OR: 0.73, 95% CI: 0.63–0.86) and 29% (OR: 0.71, 95% CI: 0.58–0.87), respectively. Regarding ever-smokers, significant associations were found between older age at menopause (OR: 0.76, 95% CI: 0.60–0.96) and younger age at first pregnancy (OR: 1.39, 95% CI: 1.05–1.85) and the risk of lung cancer (Fig. 3).

Finally, we comprehensively analyzed the risk of lung cancer in women according to both smoking status and population. Reference [29] shows that higher levels of sex steroid hormone exposure, either endogenous (OR: 0.87, 95% CI: 0.75–1.00) or exogenous (OR: 0.70, 95% CI: 0.56–0.87), decreased the risk of lung cancer in non-smoking Asian women (OR: 0.84, 95% CI: 0.74–0.96). (Supplementary Figure S4) [29].

The shape of the funnel plots (Supplementary Figure S5) [29] as well as the results of the linear regression test of funnel plot asymmetry (Supplementary Figure S6) [29] and Egger’s test (Table 2) explored the absence of publication bias. The sensitivity analysis suggested that the results were robust because the pooled ORs were not obviously changed (Supplementary Figure S7) [29].

## Discussion

Several meta-analyses on sex steroid hormones and female lung cancer risk have been published since 2009 to 2019, but several questions have not yet been answered [6, 16–28]. First, the previous meta-analysis only focused on the research on the exogenous sex steroid hormones (e.g., HRT use) with inconsistent conclusions. The comprehensive effect of both endogenous and exogenous sex steroid hormones on the risk of female lung cancer has not been investigated. Second, if effects of sex steroid hormones on the risk of female lung cancer exist, whether the effects are varied by race and biased by other factors such as tobacco smoking is unknown. Therefore, we conducted this study aiming to comprehensively explore the associations between the levels of sex steroid hormone exposure, both endogenous and exogenous, and the risk of lung cancer among women, including Asian and Western and ever-smokers and never-smokers. To our knowledge, this study updated previous meta-analyses by contributing a large quantity of new data and the biggest sample size on sex steroid hormone-related characteristics in female lung cancer patients published during 2006–2019, and this is the first study reporting the decreased risk of lung cancer in women related to the higher levels of sex steroid hormone exposure, both endogenous and exogenous.

The previous studies did not analyze the summary effect of both endogenous and exogenous sex steroid hormones on the risk of female lung cancer. One studies have reported their findings for menstrual factors [6, 16–28], two for reproductive histories [6, 16–28] and five for hormonal contraception use [6, 16–28]. Their results were not all the same: five studies indicated that increasing sex steroid hormone exposure was a protective factor for female lung cancer, and one studies have found that it was a risk factor; the remaining studies did not support the hypothesis that there was a clear link between sex steroid hormone exposure and lung cancer risk.

The levels of sex steroid hormone exposure vary by the stage of life in the Supplementary Figure S8 [29]. The production of endogenous female hormones begins increasingly growing at approximately the time of menarche and decreases rapidly until the time of menopause, and the intake of exogenous sex steroid hormones mainly depends on OC or HRT use and isoflavone intake. Women with a younger age at menarche as well as older age at menopause, longer reproductive window, longer length of each cycle and multiple pregnancies might have more ovulatory cycles and sex steroid secretion, resulting in higher cumulative levels of sex steroid hormone exposure. Additionally, the supplements of exogenous sex steroid hormones contribute to the cumulative exposure. Therefore, the effect of the cumulative exposure of sex steroid hormones on the cells should be estimated overall by combining endogenous and exogenous sex steroid hormones together. Exposure to sex steroid hormones during development results in permanent organizational effects, whereas activation effects are transient and require the continued presence of the hormone [43]. Currently, the potential mechanisms underlying the association between hormone exposure and lung cancer risk are not entirely clear; in our study they mainly involve the protective roles of sex steroid hormones in lung cancer and women. Meanwhile, animal models and functional and physiological evidence provide support for a role of sex steroid hormones in lung carcinogenesis. Estrogen, progesterone and reproductive hormones are thought to be involved in the development of lung cancer due to sex differences in the protein expression of estrogen receptor (ER)-α, ER-β, and progesterone receptor (PR) in lung cancer [16]. Localized ERs are important for alveolar formation and surfactant homeostasis in the lung, and surfactant, produced in alveolar type II cells, can clear the lungs of unwanted particles, including carcinogens [44]. A decreased risk of lung cancer was reported in ER-positive women on HRT. The use of HRT was proven to be associated with the increased levels of estrogen binding to ER-β in the lung, reducing transcription and thus reducing cell growth [45]. Some studies showed that use of HRT and a higher level of ER expression could enhance the ability of the immune system to reject malignant lung tissues early in the cancer process [46–49]. Meanwhile, the endogenous hormones, indicated by menstrual factors, might influence the role of estrogens in epithelial cell regeneration and maintenance [44]. The biology of sex steroid hormones in women is undoubtedly complex and includes enzymes involved in metabolism, receptors, regulation and crosstalk with other signaling pathways. In addition, the potential differences in the mechanisms between endogenous and exogenous hormones deserve further study.

The effects of sex steroid hormones on the risk of female lung cancer did not differ by calendar year of publication and the study design, suggesting robust associations between the decreased risk of lung cancer in women and higher levels of sex steroid hormone exposure. However, the effects vary by population and race. Older age at menopause and longer reproductive windows significantly decreased the risk of lung cancer in Western women but not in Asian women. The differences in the use of HRT between Asian and Western women might explain this finding partly. The use of HRT is more common in Western women than in Asian women, which is consistent with our findings that HRT use is a protective factor [50]. Additionally, ethnic genetic backgrounds and lifestyle differences should be also considered.

Tobacco smoking, the most established risk factor for lung cancer, confounds the effects of sex steroid hormones on the risk of female lung cancer. Peng J et al. reported enhanced estrogenic synthesis in lung cancer tissues, and their hormonal environment can synergize with the mutagenicity of tobacco smoke components [51]. Although the higher levels of sex steroid hormone exposure reduced the risk of female lung cancer in both never-smokers and ever-smokers, the association appears more pronounced for never-smokers, especially in Asians. Interestingly, older age at menopause, an indicator of lower risk of lung cancer in female ever-smokers, is an indicator for higher risk in never-smokers. This finding might be attributed to the higher proportion of never-smokers in Asian females and the less use of exogenous sex steroid hormones [6–8, 50, 52].

This study explored the correlation between sex steroid hormone exposure and the risk of lung cancer risk in women, especially in never-smoking and Asian women. We would also suggest expanding on the challenges for recruiting female non-smokers into the screening under the current screening strategy. Currently, the LDCT scan is the only official method of lung cancer screening. However, to reduce the harms of screening such as false positives, overdiagnosis and treatment, economic burden and radiation exposure, a risk assessment customized to the target population is essential. Identifying never-smokers with a cancer risk as high as those within ever-smokers who may similarly benefit from LDCT screening is an urgent matter to accommodate “equal management of people at equal risk”. This study provides evidence for exploring new risk factors of lung cancer and developing risk-assessment-based lung cancer screening strategies. This study had some limitations. First, more precise evaluation of the association, including the dose-response and a time-response relationship, could not be evaluated, due to the lack of fine data acquisition from the individual original studies, such as histologic types and hormone receptor status of lung cancer cases. Second, although the meta-analysis includes more than 30,000 female lung cancer cases and 1,400,000 subjects without lung cancer, the multiple sub-analyses are relatively less robust because of the limited ample size. Third, cut-off value for young age among the included studies was variable and not standardized. The most used cut-off value was the original definition proposed by the authors, but they varied among the included studies. Fourth, studies without original data have been excluded, which may lead to heterogeneity and possible selection bias. However, the conclusions and limitations of this study may provide some directions for the design of new trials.

## Conclusions

In summary, this meta-analysis revealed an association between higher levels of sex steroid hormone exposure and a decreased risk of female lung cancer, but the molecular biological mechanisms deserve further study. Surveillance of sex steroid hormones might be used for identifying populations at high risk of lung cancer, especially among non-smoking women. Future biological studies on the mechanism and epidemiological studies with improved design and fewer confounding factors are needed to understand the relationship between sex steroid hormones and lung cancer.

## Supplementary Information

**Additional file 1 **: **Table S1**. Search strategy (Supplementary Table S1) [29]. **Table S2**. Characteristics of included studies (Supplementary Table S2) [29]. a. 0 = Cohort, 1 = Case-control; b. 0 = Premenopausal, 1 = Postmenopausal, 2 = Premenopausal/Postmenopausal; c. 0 = Never, 1 = Ever/never; d. 0 = Adenocarcinoma, 1 = NSCLC, 2 = SCLC/NSCLC; e. 0 = Population-based controls, 1 = Hospital-based controls, 2 = population-based + hospital-based controls. **Table S3**. Quality Assessment According to the Modified Newcastle-Ottawa Scale (NOS) a (Supplementary Table S3) [29]. a. 0 = no star allocated;1 = one star allocated; 2 = two stars allocated; b. Case-control studies are scored on the exposure of interest, whereas cohort and cross-sectional studies are scored on the outcome of interest. **Figure S1**. Forest plots for high sex steroid hormone exposure on the risk of lung cancer with studies assessed both endogenous and exogenous sex steroid hormones (Supplementary Figure S1) [29]. a. OR: Odds ratio; b. CI: Confidence interval; c. OC: Oral contraception; d. HRT: Hormone replacement therapy. **Figure S2**. Forest plots for high sex steroid hormone exposure on the risk of lung cancer with (a) 1987–2007 and (b) 2008–2019 (Supplementary Figure S2) [29]. a. OR: Odds ratio; b. CI: Confidence interval; c. OC: Oral contraception; d. HRT: Hormone replacement therapy. **Figure S3**. Forest plots for high sex steroid hormone exposure on the risk of lung cancer with (a) Retrospective studies and (b) Prospective studies (Supplementary Figure S3) [29]. a. OR: Odds ratio; b. CI: Confidence interval; c. OC: Oral contraception; d. HRT: Hormone replacement therapy. **Figure S4**. Forest plots for higher levels of sex steroid hormone exposure on the risk of female lung cancer with (a) Never-smokers and (b) Ever-smokers in different ethnicities (Supplementary Figure S4) [29]. a. OR: Odds ratio; b. CI: Confidence interval. **Figure S5**. Funnel Plot (Supplementary Figure S5) [29]. **Figure S6**. Egger’s funnel plot for assessing potential publication bias (Supplementary Figure S6) [29]. **Figure S7**. Sensitivity Analysis by the Leaving-One-Out Method (Supplementary Figure S7) [29]. **Figure S8**. The levels of endogenous sex steroid hormone exposure during development among women (Supplementary Figure S8) [29]. Women, with younger age at menarche, older age at menopause, longer reproductive window, longer length of each cycle and reproductive factors, will have higher exposure of cells to endogenous sex steroid hormones over a lifetime.

## Data Availability

The dataset supporting the conclusions of this article is available in the references and the supplementary information, unique persistent identifier and hyperlink to dataset(s) in 10.6084/m9.figshare.12991559.v2.

## Abbreviations

95 % CI: 95 % confidence interval
OR: Odds ratio
OC: Oral contraceptive
HRT: Hormone replacement therapy
