# Supplementary material for: Associations between female lung cancer risk and sex steroid hormones: a systematic review and meta-analysis of the worldwide epidemiological evidence on endogenous and exogenous sex steroid hormones
Source: BMC Cancer. 2021 Jun 10;21:690. doi: 10.1186/s12885-021-08437-9 (PMC8194027; doi:10.1186/s12885-021-08437-9)
Supplement: Supplementary file 1 — Additional file 1 : Table S1. Search strategy (Supplementary Table S1) [29]. Table S2. Characteristics of included studies (Supplementary Table S2) [29]. a. 0 = Cohort, 1 = Case-control; b. 0 = Premenopausal, 1 = Postmenopausal, 2 = Premenopausal/Postmenopausal; c. 0 = Never, 1 = Ever/never; d. 0 = Adenocarcinoma, 1 = NSCLC, 2 = SCLC/NSCLC; e. 0 = Population-based controls, 1 = Hospital-based controls, 2 = population-based + hospital-based controls. Table S3. Quality Assessment According to the Modified Newcastle-Ottawa Scale (NOS) a (Supplementary Table S3) [29]. a. 0 = no star allocated;1 = one star allocated; 2 = two stars allocated; b. Case-control studies are scored on the exposure of interest, whereas cohort and cross-sectional studies are scored on the outcome of interest. Figure S1. Forest plots for high sex steroid hormone exposure on the risk of lung cancer with studies assessed both endogenous and exogenous sex steroid hormones (Supplementary Figure S1) [29]. a. OR: Odds ratio; b. CI: Confidence interval; c. OC: Oral contraception; d. HRT: Hormone replacement therapy. Figure S2. Forest plots for high sex steroid hormone exposure on the risk of lung cancer with (a) 1987–2007 and (b) 2008–2019 (Supplementary Figure S2) [29]. a. OR: Odds ratio; b. CI: Confidence interval; c. OC: Oral contraception; d. HRT: Hormone replacement therapy. Figure S3. Forest plots for high sex steroid hormone exposure on the risk of lung cancer with (a) Retrospective studies and (b) Prospective studies (Supplementary Figure S3) [29]. a. OR: Odds ratio; b. CI: Confidence interval; c. OC: Oral contraception; d. HRT: Hormone replacement therapy. Figure S4. Forest plots for higher levels of sex steroid hormone exposure on the risk of female lung cancer with (a) Never-smokers and (b) Ever-smokers in different ethnicities (Supplementary Figure S4) [29]. a. OR: Odds ratio; b. CI: Confidence interval. Figure S5. Funnel Plot (Supplementary Figure S5) [29]. Figure S6. Egger’s funnel plot fo [file 12885_2021_8437_MOESM1_ESM.docx]

**Supplementary material [29]**

**Table S1. Search strategy[53]**

| **Search strategy in PubMed**  **#1** "menarche"[tiab] OR "menstruation"[tiab] OR "menopause"[tiab] OR "menopausal"[tiab] OR "menstrual"[tiab] OR "climacteric"[tiab] OR ((menstrual[tiab] OR menstruation[tiab] OR climacteric[tiab] ) AND (histor*[tiab] OR factor*[tiab]))  **#2** "parity"[tiab] OR "gravidity"[tiab] OR "pregnancy"[tiab] OR "breastfeeding"[tiab] OR "miscarriage"[tiab] OR "abortion"[tiab] OR "fertility"[tiab] OR "age at first birth"[tiab] OR ((reproductive[tiab] OR contraceptive[tiab] OR pregnancy[tiab] OR birth[tiab] OR childbirth[tiab]) AND (histor*[tiab] OR factor*[tiab]))  **#3** "estrogens"[tiab] OR "sex hormones"[tiab] OR "oral contraceptives"[tiab] OR "OC"[tiab] OR "hormone replacement therapy"[tiab] OR "HRT"[tiab] OR "menopausal hormone therapy"[tiab] OR "menopausal hormone therapy"[tiab] OR "Isoflavone"[tiab] OR ((steroid[tiab]OR sex[tiab] OR female[tiab] OR exogenous[tiab]) AND (hormones[tiab] OR hormone[tiab]))  **#4** #1 OR #2 OR #3  **#5** (pulmonary[tiab] OR lung[tiab]) AND (adenocarcinoma*[tiab] OR adenoma*[tiab] OR cancer[tiab] OR cancers[tiab] OR carcinoma[tiab] OR carcinomas[tiab] OR neoplas*[tiab] OR tumor[tiab] OR tumors[tiab]OR tumour*[tiab])  **#6** "men"[tiab] OR "man"[tiab]  **#7** #4 AND #5 NOT #6 |
| --- |
| **Search strategy in Embase**  **#1** 'lung tumor'/exp OR 'lung cancer': ti,ab  **#2** "menarche": ti,ab OR "menstruation": ti,ab OR "menopause": ti,ab OR "menopausal”: ti,ab OR "menstrual": ti,ab OR "climacteric": ti,ab OR "parity": ti,ab OR "gravidity": ti,ab OR "pregnancy": ti,ab OR "breastfeeding": ti,ab OR "miscarriage”: ti,ab OR "abortion": ti,ab OR "fertility": ti,ab OR "age at first birth": ti,ab OR "estrogens": ti,ab OR "sex hormones": ti,ab OR "oral contraceptives": ti,ab OR "OC": ti,ab OR "hormone replacement therapy": ti,ab OR "HRT": ti,ab OR "menopausal hormone therapy": ti,ab OR "menopausal hormone therapy": ti,ab OR "Isoflavone": ti,ab  **#3** #1 AND #2 AND [female]/lim |
| **Search strategy in Web of Science**  **#1** TI = ("lung cancer" OR "lung carcinoma" OR "lung neoplasms" OR “lung tumor”)  **#2** TI = ("menarche" OR "menstruation" OR "menopause" OR "menopausal" OR "menstrual" OR "climacteric" OR "parity" OR "gravidity" OR "pregnancy" OR "breastfeeding" OR "miscarriage” OR "abortion" OR "fertility" OR "age at first birth" OR "estrogens" OR "sex hormones" OR "oral contraceptives" OR "OC" OR "hormone replacement therapy" OR "HRT" OR "menopausal hormone therapy" OR "menopausal hormone therapy" OR "Isoflavone")  **#3** "men" OR "man"  **#3** #1 AND #2 NOT #3 |
| **Search strategy in Cochrane Library**  **#1** "lung cancer": ti,ab,kw OR "lung carcinoma": ti,ab,kw OR "lung tumor": ti,ab,kw OR "lung neoplasms": ti,ab,kw  **#2** "menarche": ti,ab,kw OR "menstruation": ti,ab,kw OR "menopause": ti,ab,kw OR "menopausal": ti,ab,kw OR "menstrual": ti,ab,kw OR "climacteric": ti,ab,kw OR "parity": ti,ab,kw OR "gravidity": ti,ab,kw OR "pregnancy": ti,ab,kw OR "breastfeeding": ti,ab,kw OR "miscarriage”: ti,ab,kw OR "abortion": ti,ab,kw OR "fertility": ti,ab,kw OR "age at first birth": ti,ab,kw OR "estrogens": ti,ab,kw OR "sex hormones": ti,ab,kw OR "oral contraceptives": ti,ab,kw OR "OC": ti,ab,kw OR "hormone replacement therapy": ti,ab,kw OR "HRT": ti,ab,kw OR "menopausal hormone therapy": ti,ab,kw OR "menopausal hormone therapy": ti,ab,kw OR "Isoflavone": ti,ab,kw  **#3** "men": ti,ab,kw OR "man": ti,ab,kw  **#4** #1 AND #2 NOT #3 |

**Table S2. Characteristics of included studies**

| **Study** | **Coun-try**  **/regi-on** | **St-udy des-ign ^a^** | **Stu-dy peri-od** | **Study Population** | | | **Cases** | | **Control** | | **Details of exposure (case/control)** |
| --- | --- | --- | --- | --- | --- | --- | --- | --- | --- | --- | --- |
|  |  |  |  | **Age range /mean± SD (Case/ control)** | **Menopausal status ^b^** | **Smoking status ^c^** | **n** | **Histologic type of cases ^d^** | **n** | **Comparison group ^e^** |  |
| Ashley  et al, 2019[54] | United States | 0 | 1993-2009 | 50-78 | 2 | 1 | 1147 | 1 | 1147 | 0 | history of use of HRT (685/458) |
| Jin  et al, 2019[33] | China | 1 | 2003-2010 | 66.79± 9.36/ 67.37±9.32 | 2 | 1 | 680 | N.A. | 1808 | 0 | younger age at menarche: ≤ 17 (224/639); older age at menopause: ≥ 46 (568/1516); longer reproductive windows: ≥ 33 (407/1057); multiple pregnancies: ≥ 2 (561/1582); history of use of OC (23/93) |
| Vohra  et al, 2019[55] | Nepal | 1 | 2009-2012 | 23-85 | 2 | 1 | 267 | N.A. | 226 | 1 | younger age at menarche: ≤ 14 (104/442); older age at menopause: ≥ 45 (169/107); history of pregnancy (227/196); younger age at first pregnancy: ≤ 20 (150/127); multiple pregnancies: ≥ 3 (227/183); history of use of OC (29/39) |
| He  et al, 2017[56] | China | 1 | 2006-2015 | 56.44±10.83/  56.51±10.72 | 2 | 1 | 477 | 2 | 479 | 1 | younger age at menarche: ≤ 12 (46/25); older age at menopause: ≥ 51 (121/97); younger age at first pregnancy: ≤ 24 (323/224); multiple pregnancies: ≥ 3 (210/144); history of use of OC (49/35); history of use of HRT (33/24) |
| Schwartz  et al, 2015[57] | United States | 0 | 1993-2012 | 50-79 | 2 | 1 | 2467 | 2 | 158388 | 0 | younger age at menarche: ≤ 13 (1869/587); older age at menopause: ≥ 50 (1020/1208); history of pregnancy (2247/211); younger age at first pregnancy: ≤ 19 (379/20065); multiple pregnancies: ≥ 3 (1647/600); history of use of OC (946/1521); history of use of HRT (1442/3492) |
| Brittany  et al, 2014[58] | United States | 0 | 1976-1982 | 30-55 | 1 | 1 | 2644 | N.A. | 118933 | 0 | history of use of OC (1007/1637) |
| Brenner  et al, 2013[25] | Germany | 1 | 2002-2003 | N.A. | 2 | N.A. | 52 | 2 | 52 | 0 | history of use of OC (33/33); history of use of HRT (26/25) |
| Chen  et al, 2013[59] | China | 1 | 2002-2009 | 60.54/ 60.41 | 2 | 0 | 532 | 0 | 532 | 1 | multiple pregnancies: ≥ 2 (487/481) |
| Gallagher  et al, 2013[60] | China | 0 | 1989-2000 | N.A. | 2 | 1 | 824 | N.A. | 266576 | 0 | history of pregnancy (569/33); multiple pregnancies: ≥ 3 (400/169) |
| Lo  et al, 2013[61] | China | 1 | 2002-2009 | 58.78± 11.33 /58.25 ±11.29 | 2 | 0 | 1221 | 2 | 1221 | 1 | history of use of OC (166/141); history of use of HRT (272/337) |
| Pesatori  et al, 2013[62] | Italy | 1 | 2002-2005 | 35-79 | 2 | 1 | 407 | 2 | 499 | 0 | younger age at menarche: ≤ 13 (336/424); older age at menopause: ≥ 46 (214/312); longer reproductive windows: ≥ 33 (237/348); longer menstrual cycle: ≥ 27 (313/370); history of pregnancy (329/411); younger age at first pregnancy: ≤ 25 (170/174); multiple pregnancies: ≥ 2 (217/294); history of use of OC (101/132); history of use of HRT (63/112) |
| Brinton  et al, 2012[63] | United States | 0 | 1995-2006 | 50-71 | N.A. | 1 | 2097 | N.A. | 115911 | 0 | history of use of HRT (1002/1539) |
| Lim  et al, 2012[34] | Singapore | 1 | 1996-1998/ 2005-2008 | 65.9/64.1 | 2 | 1 | 702 | 2 | 1578 | 1 | younger age at menarche: ≤ 14 (106/274); older age at menopause: ≥ 49 (377/779); longer reproductive windows: ≥ 34 (358/757); longer menstrual cycle: ≥ 31 (44/159); history of pregnancy (614/1399); younger age at first pregnancy: ≤ 25 (410/950); multiple pregnancies: ≥ 3 (427/1016) |
| Yang  et al, 2012[64] | China | 0 | 1997-2008 | 40-70 | 2 | 1 | 370 | N.A. | 71180 | 0 | higher isoflavone intake from food (57/279) |
| Brinton  et al, 2011[37] | United States | 0 | 1995-1996 | 50-71 | 2 | 1 | 3512 | 2 | 181505 | 0 | younger age at menarche: ≤ 14 (3164/331); history of pregnancy (1620/372); younger age at first pregnancy: ≤ 24 (2330/111414); multiple pregnancies: ≥ 3 (1806/1207); history of use of OC (1701/1715); history of use of HRT (1698/1808) |
| Meinhold  et al, 2011[35] | United States | 1 | 1998-2008 | 66/64 | 2 | 1 | 430 | 1 | 611 | 2 | younger age at menarche: ≤ 13 (308/445); older age at menopause: ≥ 50 (97/180); history of pregnancy (385/552); younger age at first pregnancy: ≤ 24 (295/432); multiple pregnancies: ≥ 3 (1777/298); history of use of OC (196/285); history of use of HRT (194/311) |
| Shimazu  et al, 2011[65] | Japan | 0 | 1990-2006 | 40-69 | 2 | 1 | 126 | 2 | 252 | 0 | history of use of HRT (19/26); higher isoflavone intake from food (96/202) |
| Christopher et al, 2010[66] | United States | 0 | 2000-2008 | 50-76 | 1 | 1 | 344 | 2 | 34244 | 0 | history of use of HRT (230/104) |
| Paulus  et al, 2010[38] | United States | 1 | 1992-2003 | 66.2/58.4 | N.A. | 1 | 1004 | 1 | 848 | 2 | history of pregnancy (534/659); younger age at first pregnancy: ≤ 24 (358/398); multiple pregnancies: ≥ 2 (470/585) |
| Shimazu  et al, 2010[42] | Japan | 0 | 1995-2005 | 45-75 | 2 | 1 | 178 | N.A. | 40306 | 0 | higher isoflavone intake from food (129/49) |
| Chlebowski  et al, 2009[67] | United States | 0 | 1993-2005 | 50-79 | 2 | 1 | 194 | 2 | 16414 | 0 | history of use of HRT (109/85) |
| Dorjgochoo  et al, 2009[68] | China | 0 | 1997-2000 | 40-70 | 2 | 1 | 229 | N.A. | 66432 | 0 | history of use of OC (44/185) |
| Koushik  et al,2009[69] | Canada | 1 | 1996-1997 | 61.5 | 2 | 1 | 422 | 2 | 577 | 0 | younger age at menarche: ≤ 15 (278/469); older age at menopause: ≥ 45 (168/296); history of pregnancy (343/470); younger age at first pregnancy: ≤ 24 (235/260); multiple pregnancies: ≥ 2 (277/364) |
| Seow  et al, 2009[70] | Singapore | 0 | 1993-2005 | 45-74 | 2 | 1 | 298 | N.A. | 35000 | 0 | higher isoflavone intake from food (122/176) |
| Mahabir  et al, 2008[71] | United States | 1 | 1995-2005 | 60.75/60.11 | N.A. | 1 | 763 | N.A. | 838 | 1 | history of use of HRT (220/231) |
| Rodriguez  et al, 2008[39] | United States | 0 | 1992-2003 | 50-74 | 2 | 1 | 659 | 2 | 72772 | 0 | history of use of HRT (355/304) |
| Chen  et al, 2007[72] | China | 1 | 2002-2006 | 59.73(12.30)/  57.40(14.16) | 2 | 1 | 826 | 2 | 531 | 1 | Menopause (177/144); history of use of OC (56/47); history of use of HRT (145/134) |
| Hannaford  et al, 2007[73] | United Kingdom | 0 | 1968-1996 | 29±6.6 | 0 | 1 | 297 | N.A. | 45703 | 0 | history of use of OC (206/91) |
| Kabat  et al, 2007[74] | Canada | 0 | 1980-2000 | 40-59 | 2 | 1 | 750 | 2 | 89062 | 0 | history of use of OC (417/52101); history of use of HRT(211/17794) |
| Matsuo  et al, 2007[75] | Japan | 1 | 2000-2005 | 18-79 | 2 | 1 | 173 | 1 | 861 | 1 | older age at menopause: ≥ 45 (642/126); longer reproductive windows: ≥ 35 (102/505); younger age at first pregnancy: ≤ 24 (13/83) |
| Ramnath  et al, 2007[76] | United States | 1 | 1982-1998 | 61 | N.A. | 1 | 595 | N.A. | 1195 | 1 | history of use of OC (132/243); history of use of HRT (132/338) |
| Schwartz  et al, 2007[77] | United States | 1 | 2001-2005 | 18-74 | 2 | 1 | 488 | 1 | 498 | 0 | history of pregnancy (44/97); multiple pregnancies: ≥ 3 (434/419); history of use of OC (253/208); history of use of HRT (350/367) |
| Elliott  et al, 2006[78] | United Kingdom | 1 | 1968-2004 | 29 | N.A. | 1 | 162 | N.A. | 486 | 0 | history of use of OC (67/187); history of use of HRT (13/42) |
| Gorlova  et al, 2006[79] | United States | 1 | 1995-2003 | 60.2±12.7/  61.9±10.9 | 2 | 0 | 280 | 2 | 242 | 1 | history of pregnancy (172/160); history of use of OC (4/4); history of use of HRT (75/73) |
| Liu  et al, 2005[80] | Japan | 0 | 1990-2002 | 40-69 | 2 | 1 | 153 | 2 | 58950‬ | 0 | younger age at menarche: ≤ 13 (23/123); older age at menopause: ≥ 46 (92/26); longer reproductive windows: ≥ 31 (74/31); younger age at first pregnancy: ≤ 25 (78/25212); multiple pregnancies: ≥ 3 (81/56) |
| Schabath  et al, 2005[41] | United States | 1 | 1995-2003 | 62.1±10.3/  61.5±9.4 | N.A. | 1 | 774 | N.A. | 848 | 0 | higher isoflavone intake from food (172/212) |
| Schabath  et al, 2004[40] | United States | 1 | N.A. | 59.7/58.6 | 2 | 1 | 499 | N.A. | 519 | 1 | history of use of OC (9/12); history of use of HRT (232/273) |
| Brenner  et al, 2003[81] | China | 1 | 1994-1998 | 30-75 | 2 | 1 | 118 | 2 | 435 | 0 | younger age at menarche: ≤ 16 (79/300); older age at menopause: ≥ 45 (48/230); longer reproductive windows: ≥ 34 (25/134); longer menstrual cycle: ≥ 31 (82/319); history of pregnancy (108/431); younger age at first pregnancy: ≤ 18 (22/124); multiple pregnancies: ≥ 3 (85/357) |
| Kreuzer  et al, 2003[82] | Germany | 1 | 1990-1996 | 60/59 | 2 | 1 | 811 | 2 | 912 | 0 | younger age at menarche: ≤ 14 (546/644); older age at menopause: ≥ 50 (216/318); longer menstrual cycle: ≥ 28 (594/711); history of pregnancy (673/779); younger age at first pregnancy: ≤ 21 (286/223); multiple pregnancies: ≥ 2 (453/551); history of use of OC (279/354); history of use of HRT (196/274) |
| Zatloukal  et al, 2003[83] | Czech | 1 | 1998-2002 | 25-89 | 2 | 1 | 366 | 2 | 1624 | 1 | younger age at menarche: ≤ 14 (262/1235); longer menstrual cycle: ≥ 30 (29/253) |
| Blackman  et al, 2002[84] | United States | 1 | 1976-2001 | 40-74 | 2 | 1 | 662 | 2 | 4671 | 1 | history of use of HRT (89/379) |
| Hulley  et al, 2002[85] | United States | 0 | 1993-2000 | 67 | 1 | 1 | 64 | N.A. | 5020 | 0 | history of use of HRT (37/27) |
| Seow  et al, 2002[36] | Singapore | 1 | 1996-1998 | N.A. | 2 | 1 | 303 | 2 | 765 | 1 | younger age at menarche: ≤ 13 (277/113); longer menstrual cycle: ≥ 31 (29/113); history of pregnancy (261/684); younger age at first pregnancy: ≤ 19 (54/135); multiple pregnancies: ≥ 3 (191/511); higher isoflavone intake from food (121/176) |
| Baosen  et al, 2000[86] | China | 1 | 1991-1995 | 35-69 | N.A. | 0 | 72 | 0 | 72 | 0 | younger age at menarche: ≤ 16 (34/42); longer menstrual cycle: ≥ 30 (61/62); multiple pregnancies: ≥ 3 (61/55) |
| Taioli  et al, 1994[87] | United States | 1 | N.A. | N.A. | N.A. | 1 | 180 | 0 | 303 | 1 | younger age at menarche: ≤ 15 (134/217); longer menstrual cycle: ≥ 29 (130/215); history of pregnancy (163/259); younger age at first pregnancy: ≤ 19 (30/49); multiple pregnancies: ≥ 2 (145/216) |
| Wu-Williams  et al, 1990[88] | China | 1 | 1985-1987 | 55.9/55.4 | 2 | 1 | 965 | 2 | 959 | 0 | younger age at menarche: ≤ 13 (55/64); older age at menopause: ≥ 50 (309/355); multiple pregnancies: ≥ 3 (763/746); history of use of OC (54/68) |
| Wu  et al, 1988[89] | United States | 1 | 1983-1986 | 30-75 | 2 | 1 | 336 | 0 | 257 | 0 | younger age at menarche: ≤ 14 (283/284); older age at menopause: ≥ 49 (90/115); history of use of OC (70/85); history of use of HRT (149/134) |
| Gao  et al, 1987[17] | China | 1 | 1984-1986 | 35-69 | N.A. | 1 | 672 | 2 | 735 | 0 | longer menstrual cycle: ≥ 26 (556/655) |
| a. 0 = Cohort, 1 = Case-control; b. 0 = Premenopausal, 1 = Postmenopausal, 2 = Premenopausal/Postmenopausal; c. 0 = Never, 1 = Ever/never; d. 0 = Adenocarcinoma, 1 = NSCLC, 2 = SCLC/NSCLC; e. 0 = Population-based controls, 1 = Hospital-based controls, 2 = population-based + hospital-based controls. | | | | | | | | | | | |

**Table S3. Quality Assessment According to the Modified Newcastle-Ottawa Scale (NOS) ^a^**

| **Study** | **Selection** | | | **Comparability** | | | Exposure/Outcome **^b^** | | | **Overall Score** | **Risk of Bias** | **Outcome measurement method** |
| --- | --- | --- | --- | --- | --- | --- | --- | --- | --- | --- | --- | --- |
|  | **S1** | **S2** | **S3** | **S4** | **C1** | **C2** | **E1/O1** | **E2/O2** | **E3/O3** | **(Max. of 9)** |  |  |
| Ashley et al, 2019[54] | 1 | 1 | 1 | 1 | 1 | 1 | 1 | 1 | 0 | 8 | low | Combination |
| Jin et al, 2019[33] | 1 | 1 | 1 | 1 | 1 | 1 | 1 | 1 | 0 | 8 | low | Medical record |
| Vohra et al, 2019[55] | 1 | 1 | 0 | 1 | 1 | 1 | 1 | 1 | 0 | 7 | low | Medical record |
| He et al, 2017[56] | 1 | 1 | 0 | 1 | 1 | 1 | 1 | 1 | 0 | 7 | low | Medical record |
| Schwartz et al, 2015[57] | 1 | 1 | 1 | 1 | 1 | 1 | 1 | 1 | 0 | 8 | low | Medical record |
| Brittany et al, 2014[58] | 0 | 1 | 1 | 1 | 1 | 1 | 1 | 1 | 0 | 7 | low | Combination |
| Brenner et al, 2013[25] | 0 | 1 | 1 | 1 | 1 | 1 | 1 | 1 | 0 | 7 | low | Registry-linkage |
| Chen et al, 2013[59] | 1 | 1 | 1 | 1 | 1 | 1 | 1 | 1 | 0 | 8 | low | Medical record |
| Gallagher et al, 2013[60] | 0 | 1 | 1 | 1 | 1 | 1 | 1 | 1 | 0 | 7 | low | Combination |
| Lo et al, 2013[61] | 1 | 1 | 0 | 1 | 1 | 1 | 1 | 1 | 0 | 7 | low | Medical record |
| Pesatori et al, 2013[62] | 1 | 1 | 1 | 1 | 1 | 1 | 1 | 1 | 0 | 8 | low | Medical record |
| Brinton et al, 2012[63] | 1 | 1 | 0 | 1 | 1 | 1 | 1 | 1 | 0 | 7 | low | Registry-linkage |
| Lim et al, 2012[34] | 1 | 1 | 0 | 1 | 1 | 1 | 1 | 1 | 0 | 7 | low | Medical record |
| Yang et al, 2012[64] | 1 | 1 | 1 | 1 | 1 | 1 | 1 | 1 | 0 | 8 | low | Combination |
| Brinton et al, 2011[37] | 1 | 1 | 0 | 1 | 1 | 1 | 1 | 1 | 0 | 7 | low | Registry-linkage |
| Meinhold et al, 2011[35] | 1 | 1 | 0 | 1 | 1 | 1 | 1 | 1 | 0 | 7 | low | Medical record |
| Shimazu et al, 2011[65] | 1 | 1 | 0 | 1 | 1 | 1 | 1 | 1 | 0 | 7 | low | Combination |
| Christopher et al, 2010[66] | 1 | 1 | 1 | 1 | 0 | 0 | 1 | 1 | 0 | 6 | low | Registry-linkage |
| Paulus et al, 2010[38] | 1 | 1 | 0 | 1 | 1 | 1 | 1 | 1 | 0 | 7 | low | Medical record |
| Shimazu et al, 2010[42] | 1 | 1 | 0 | 1 | 1 | 1 | 1 | 1 | 0 | 7 | low | Combination |
| Chlebowski et al, 2009[67] | 1 | 1 | 1 | 1 | 1 | 1 | 1 | 1 | 0 | 8 | low | Combination |
| Dorjgochoo et al, 2009[68] | 1 | 1 | 1 | 1 | 1 | 1 | 1 | 0 | 1 | 8 | low | Medical record |
| Koushiket al,2009[69] | 1 | 1 | 1 | 1 | 1 | 1 | 1 | 1 | 0 | 8 | low | Combination |
| Seow et al, 2009[70] | 0 | 1 | 1 | 1 | 1 | 1 | 1 | 1 | 0 | 7 | low | Medical record |
| Mahabir et al, 2008[71] | 1 | 1 | 0 | 1 | 1 | 1 | 1 | 1 | 0 | 7 | low | Medical record |
| Rodriguez et al, 2008[39] | 1 | 1 | 0 | 1 | 1 | 1 | 1 | 1 | 0 | 7 | low | Combination |
| Chen et al, 2007[72] | 1 | 1 | 0 | 1 | 1 | 1 | 1 | 1 | 0 | 7 | low | Medical record |
| Hannaford et al, 2007[73] | 1 | 1 | 1 | 1 | 1 | 1 | 1 | 1 | 0 | 8 | low | Combination |
| Kabat et al, 2007[74] | 1 | 1 | 0 | 1 | 1 | 1 | 1 | 1 | 0 | 7 | low | Registry-linkage |
| Matsuo et al, 2007[75] | 1 | 1 | 0 | 1 | 1 | 1 | 0 | 1 | 0 | 6 | low | Medical record |
| Ramnath et al, 2007[76] | 0 | 1 | 0 | 1 | 1 | 1 | 1 | 1 | 0 | 6 | low | Registry-linkage |
| Schwartz et al, 2007[77] | 0 | 1 | 1 | 1 | 1 | 1 | 1 | 1 | 0 | 7 | low | Registry-linkage |
| Elliott et al, 2006[78] | 0 | 1 | 1 | 1 | 1 | 1 | 1 | 1 | 0 | 7 | low | Registry-linkage |
| Gorlova et al, 2006[79] | 1 | 0 | 0 | 1 | 1 | 1 | 0 | 1 | 1 | 6 | low | Medical record |
| Liu et al, 2005[80] | 1 | 1 | 0 | 1 | 1 | 1 | 1 | 1 | 0 | 7 | low | Combination |
| Schabath et al, 2005[41] | 1 | 1 | 0 | 1 | 1 | 1 | 1 | 1 | 1 | 8 | low | Medical record |
| Schabath et al, 2004[40] | 1 | 1 | 0 | 1 | 1 | 1 | 0 | 1 | 1 | 7 | low | Combination |
| Brenner et al, 2003[81] | 1 | 1 | 1 | 1 | 1 | 1 | 1 | 1 | 0 | 8 | low | Medical record |
| Kreuzer et al, 2003[82] | 1 | 1 | 1 | 1 | 1 | 1 | 1 | 1 | 0 | 8 | low | Medical record |
| Zatloukal et al, 2003[83] | 1 | 1 | 0 | 1 | 1 | 1 | 0 | 1 | 0 | 6 | low | Medical record |
| Blackman et al, 2002[84] | 1 | 1 | 0 | 1 | 1 | 1 | 1 | 1 | 0 | 7 | low | Medical record |
| Hulley et al, 2002[85] | 0 | 1 | 1 | 1 | 1 | 1 | 1 | 0 | 1 | 7 | low | Medical record |
| Seow et al, 2002[36] | 1 | 1 | 0 | 1 | 1 | 1 | 0 | 1 | 0 | 6 | low | Medical record |
| Baosen et al, 2000[86] | 1 | 1 | 1 | 1 | 1 | 0 | 1 | 1 | 1 | 8 | low | Medical record |
| Taioli et al, 1994[87] | 1 | 1 | 0 | 1 | 1 | 1 | 1 | 1 | 0 | 7 | low | Medical record |
| Wu-Williams et al, 1990[88] | 1 | 1 | 1 | 1 | 1 | 0 | 0 | 1 | 0 | 6 | low | Medical record |
| Wu et al, 1988[89] | 0 | 1 | 1 | 1 | 1 | 1 | 1 | 1 | 0 | 7 | low | Registry-linkage |
| Gao et al, 1987[17] | 1 | 1 | 1 | 1 | 1 | 1 | 1 | 1 | 0 | 8 | low | Combination |
| a. 0 = no star allocated;1 = one star allocated; 2 = two stars allocated; b. Case-control studies are scored on the exposure of interest, whereas cohort and cross-sectional studies are scored on the outcome of interest. | | | | | | | | | | | | |

**Figure S1. Forest plots for the subgroup analysis by sources of sex steroid hormones**


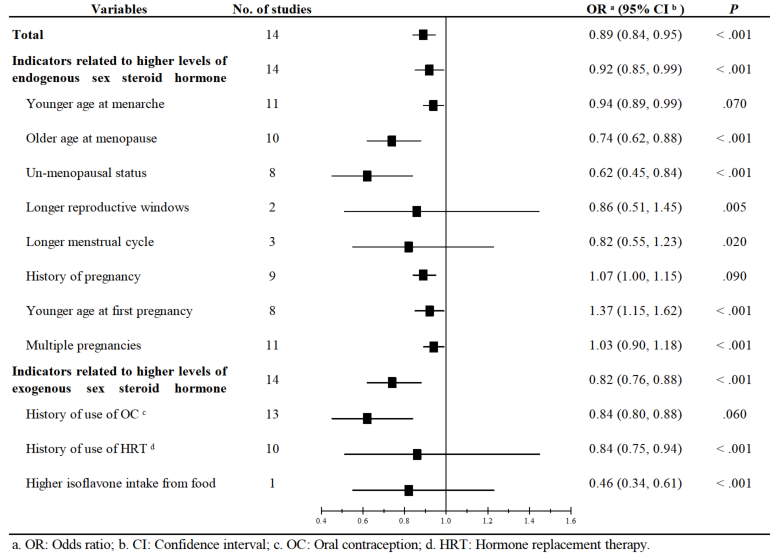


**Figure S2. Forest plots for the subgroup analysis by calendar year of publication**


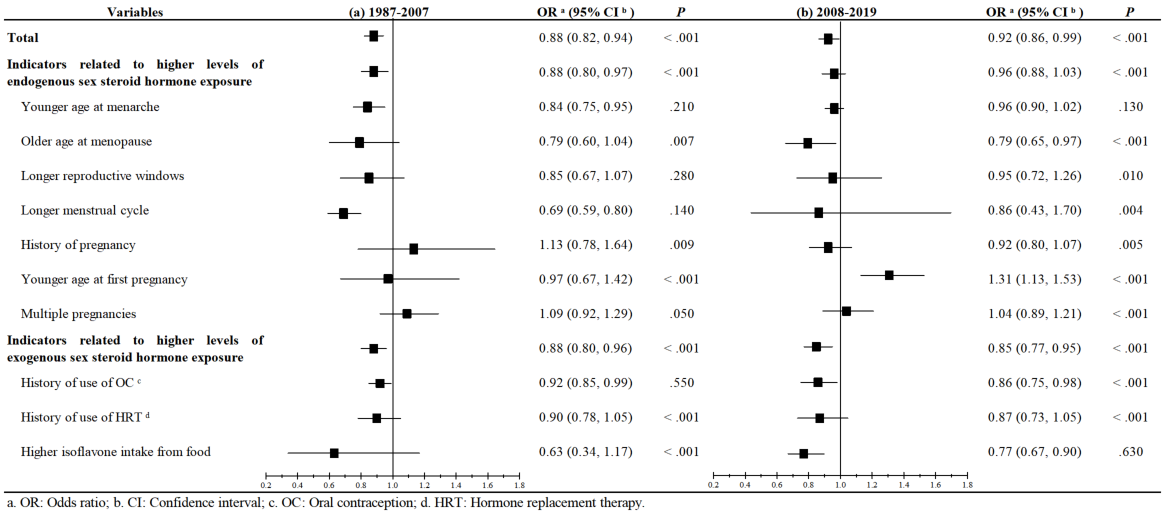


**Figure S3. Forest plots for the subgroup analysis by study design**


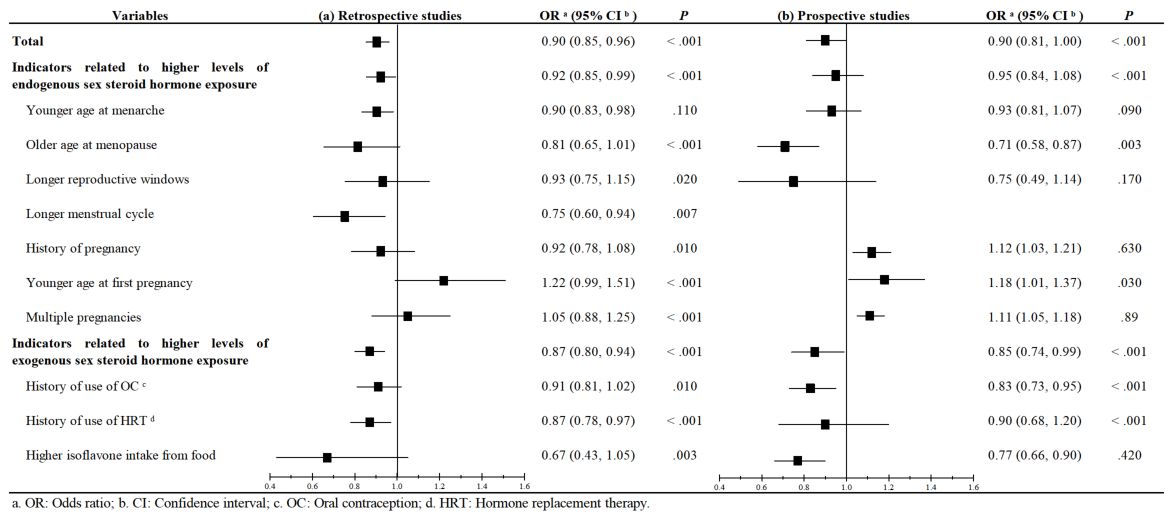


**Figure S4. Forest plots for the subgroup analysis by population and smoking status**

**
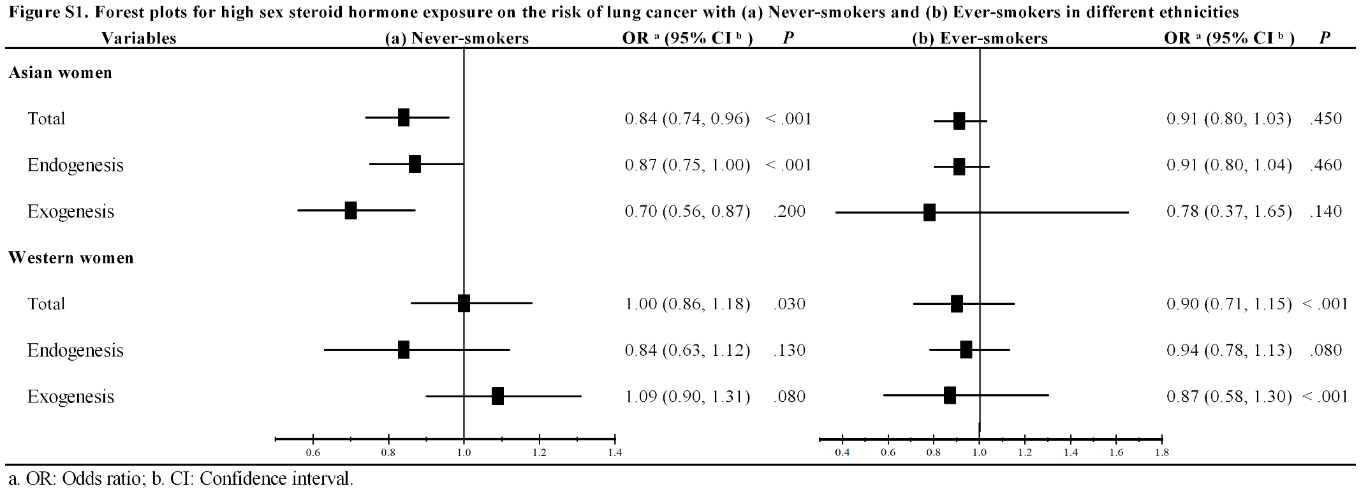
**

**Figure S5. Funnel Plot**

**
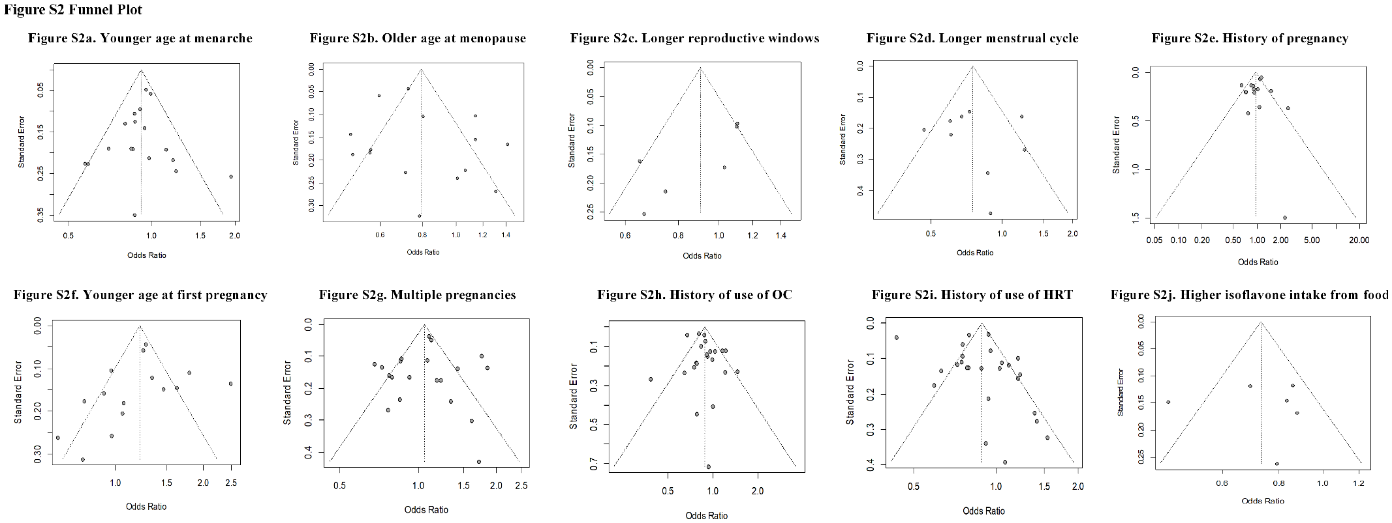
**

**Figure S6. Egger’s funnel plot for assessing potential publication bias**

**
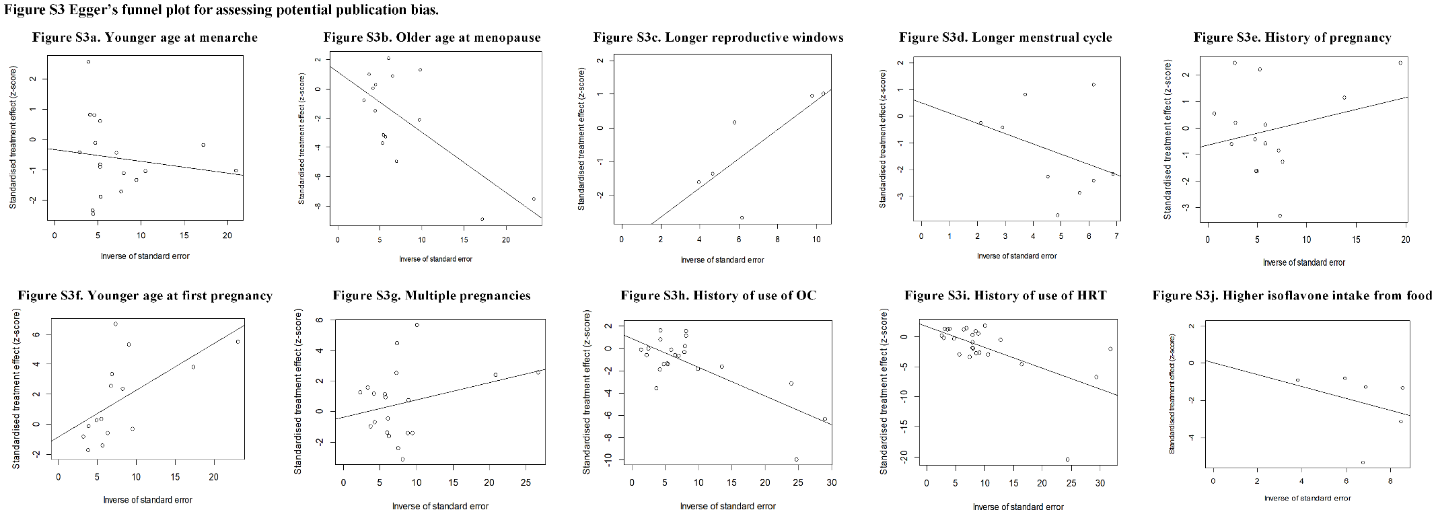
**

**Figure S7. Sensitivity Analysis by the Leaving-One-Out Method**

**
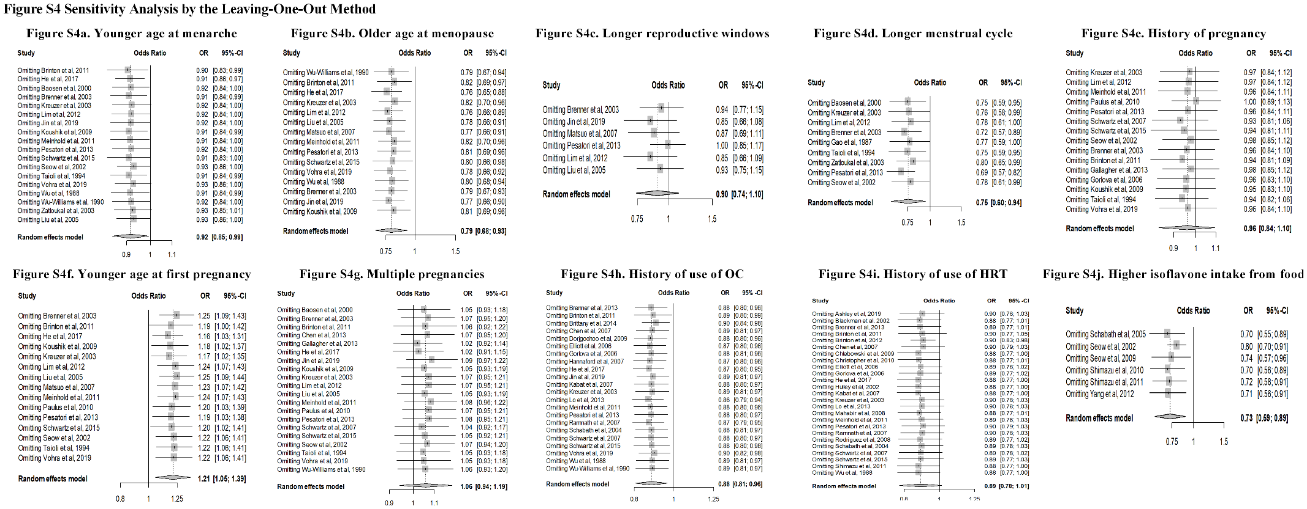
**

**Figure S8. The levels of endogenous sex steroid hormone exposure during development among women**

**
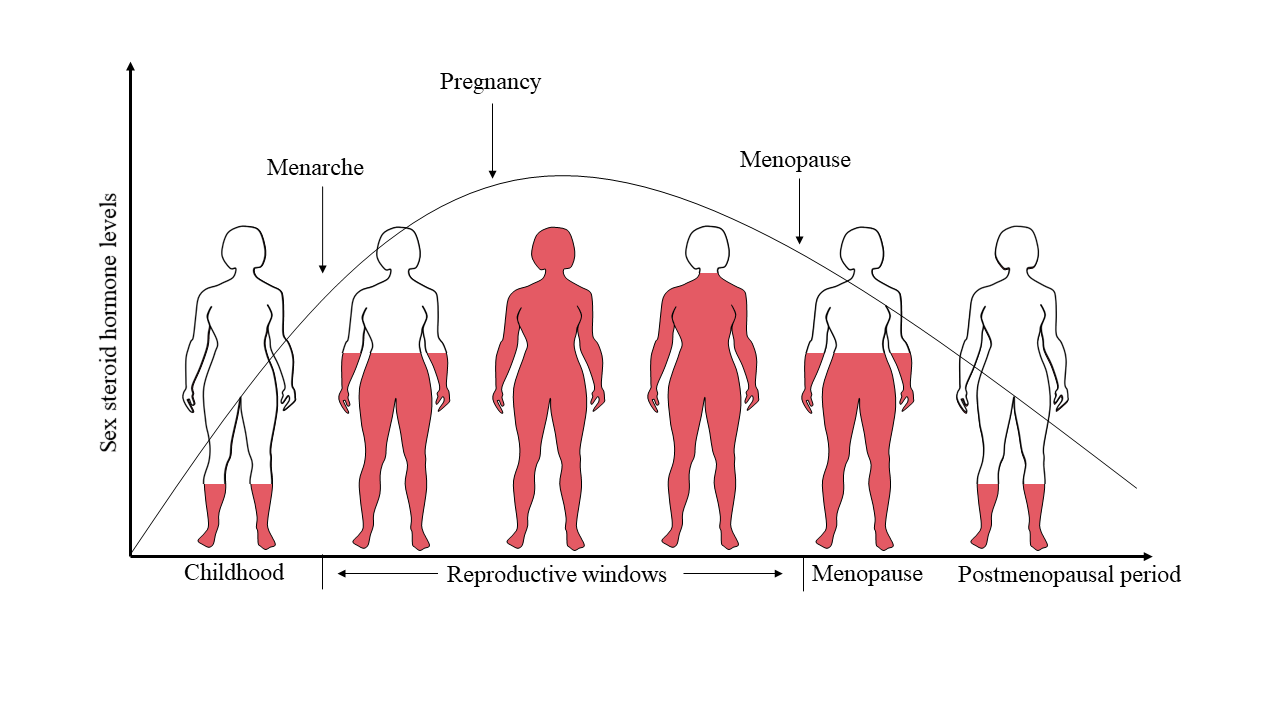
**

Women, with younger age at menarche, older age at menopause, longer reproductive window, longer length of each cycle and reproductive factors, will have higher exposure of cells to endogenous sex steroid hormones over a lifetime.
